# Factors Associated with the Acceptability of Male Circumcision among Men in Jamaica

**DOI:** 10.1371/journal.pone.0075074

**Published:** 2013-09-16

**Authors:** Melonie M. Walcott, Pauline E. Jolly, John E. Ehiri, Ellen Funkhouser, Mirjam C. Kempf, Deborah Hickman, Maung Aung, Kui Zhang

**Affiliations:** 1 Department of Epidemiology, University of Alabama at Birmingham, Birmingham, Alabama, United States of America; 2 Division of Health Promotion Sciences, University of Arizona, Tucson, Arizona, United States of America; 3 Division of Preventive Medicine, University of Alabama at Birmingham, Birmingham, Alabama, United States of America; 4 Department of Family/Child Health and Caregiving and Department of Health Behavior, University of Alabama at Birmingham, Birmingham, Alabama, United States of America; 5 Epidemiology Unit, Western Regional Health Authority, Jamaica; 6 Department of Biostatistics, University of Alabama at Birmingham, Birmingham, Alabama, United States of America; University of Washington, United States of America

## Abstract

**Objectives:**

To determine the prevalence of male circumcision (MC) among men in the western region of Jamaica, and to identify factors associated with acceptability of MC for self, infants (<1 year) and older sons (1-17 years).

**Methods:**

A cross-sectional, interviewer-administered questionnaire survey of 549 men aged 19-54 years was conducted in the western region of Jamaica. The survey included questions about the acceptance of MC for self, infants, and sons before and after an information session about the benefits of MC in preventing HIV/STI transmission. Logistic regression models were used to identify factors that were associated with acceptability of MC. Adjusted odds ratios (AOR) and 95% confidence intervals (CI) were calculated from the models.

**Results:**

Fourteen percent of the men reported that they were circumcised. In the multivariable model, which adjusted for age, education, religion and income, there were increased odds of accepting MC for infants/sons among uncircumcised men who accepted MC for self (AOR=8.1; 95% CI = 4.1-15.9), believed they would experience more pleasure during sex if circumcised (AOR=4.0; 95% CI = 2.0-8.2), and reported having no concerns regarding MC (AOR=3.0; 95% CI = 1.8-4.8). Similarly, uncircumcised men who reported no concerns about MC or who believed that they would experience more pleasure during sex if circumcised were more likely to accept MC for self.

**Conclusion:**

Providing men with information about MC increased acceptance of MC for self, infants (<17 years) and sons (1-17 years). Since targeted education on the benefits of male circumcision for prevention of HIV/STI can be effective in increasing acceptability of MC, health professionals should be trained, and willing to discuss MC with men in healthcare facilities and in the community.

## Introduction

In 2007, the World Health Organization (WHO) and the United Nations Program on HIV/AIDS (UNAIDS) recommended the inclusion of male circumcision (MC) in HIV prevention programs, especially in countries with generalized heterosexual HIV epidemics, high HIV prevalence, and low prevalence of MC [1-4]. This recommendation was based on epidemiological evidence which shows that MC is effective in reducing HIV acquisition by approximately 60% among males during heterosexual sex [1,5-9]. The WHO/UNAIDS and United Nations Children’s Fund (UNICEF) also recommend the implementation of early infant male circumcision to sustain gains made in averting new HIV infections among adults [10]. Ideally, MC should be offered to infant and adult males up to 35 years of age [11]. MC among infants is more cost effective, simpler, and less likely to result in adverse events [10,12-15]. Countries are often faced with decisions regarding the inclusion of MC in their HIV prevention package as well as whether to offer MC to infants, adults, or both, in light of resource constraints, cultural and gender norms, and religious beliefs.

Further, progress has been slow to include MC as part of the comprehensive HIV package in many countries due to challenges such as lack of political will, reports of adverse events, low acceptability of MC in some instances [3,16], and disagreement among health workers and policy makers about the benefits of MC [17]. These challenges threaten the potential impact of MC to reduce the incidence of HIV, since high uptake is required to substantially reduce transmission of the infection [7]. Major barriers to the acceptance of MC include concerns about pain during and after the procedure, cost, fear of complications and adverse events such as bleeding, adhesion, and infections [7,18,19]. Factors such as perceptions of improved penile hygiene [20-22], reduced risk of infection (including HIV/STIs) [7,20,22,23], and beliefs that MC will increase sexual pleasure [22,24] have been identified as facilitators of MC.

The Caribbean region could significantly benefit from MC as the HIV prevalence in the region is second highest to sub-Saharan Africa [17,25,26], and the primary route of HIV transmission is through heterosexual sex [27]. However, MC is not included in the regions’ HIV prevention program due to factors such as disagreement among policy makers and health professionals about the benefits of MC, concerns about the effect of MC on sexual performance, and a general misunderstanding of the role of MC as an HIV prevention strategy [17]. It is particularly important to strengthen HIV prevention strategies with non-behavioral approaches in the Caribbean due to pervasive gender norms that stigmatize homosexuality, foster dominance over females, and encourage men to have multiple sexual partners [28,29]. Together, these increase men’s vulnerability to HIV infection.

Two studies conducted by Brito et al in the Dominican Republic concluded that men are more likely to accept MC if well informed about its benefits [21,30]. In 2008, Figueroa conducted a study involving 143 STI clinic attendees in Jamaica and found that while infant MC is likely to be supported, adult male circumcision may not be feasible due to resource constraints, relatively low HIV prevalence (1.7%) and unlikely support from policy makers, surgeons and men [31]. In Figueroa’s study, only 9% of the males reported that they were circumcised, 23% of the men stated that they would consider MC, while 38% would recommend it to their sons [31]. Despite the useful data provided by Figueroa’s study, the factors that are associated with acceptance of MC among men in Jamaica (a non-traditional MC country) are still unknown. There is also the likelihood that HIV prevalence is underestimated in Jamaica due to low rates of HIV testing among adults [32,33]. Additionally, Jamaican men tend to engage in more risky sexual behaviors compared to women [27,34], with a high percentage (≥50%) of men reporting multiple sexual partners in the past 12 months [27,32,34,35]. Multiple sexual partners is a major risk factor for HIV acquisition in Jamaica as 80% of the reported AIDS cases among adults had a history of having multiple sex partners [27]. Although men tend to engage in more high risk sexual behaviors they are also less likely to be tested for HIV [27,32,33]. Thus, MC, a highly efficacious intervention which is not dependent on behavior modification would be important given the existing risk factors for HIV acquisition in Jamaica. The present study among men in the western region of Jamaica ascertained: (1) the prevalence of MC, (2) the awareness of MC, (3) the acceptance of MC for self, infants (<1 year), older sons (1-17 years), and (4) factors associated with each (prevalence, awareness, and acceptance).

## Materials and Methods

### Ethics Statement

Ethical approval for the study was obtained from the Institutional Review Board of the University of Alabama at Birmingham, the Advisory Panel of Ethics and Medico-Legal Affairs in the Jamaican Ministry of Health, and the Western Regional Health Authority. Written informed consent was obtained from each participant as approved by all the ethics review boards.

### Study design and settings

A cross-sectional questionnaire survey was conducted between June and August 2011 among 549 men aged 19-54 years in western Jamaica. Participants were recruited from the four government hospitals under the Western Regional Health Authority (WRHA) that serves the four parishes of St. James, Hanover, Westmoreland, and Trelawny, encompassing a population of 474,944 [36]. The population served by the hospitals in each parish is as follows: St. James -182,600 (39%), Westmoreland -141,800 (31%), Trelawny -72,500 (16%), Hanover -67,200 (14%). We established quotas for the number of participants to be recruited from each hospital based on the percentages of the population represented in each parish. In our sample, 34.6% of the participants were recruited form St James, 32.6% Westmoreland, 17% Trelawny, and 15.7% from Hanover. The parishes in the WRHA have high rates of HIV prevalence with St. James having the highest cumulative number of AIDS cases of the 14 parishes in Jamaica [27,37]. Health care services were available at no cost at the 4 hospitals at the time this study was conducted.

### Participants

To be eligible for participation in this study the men had to be aged 19-54 years, live in the western region of Jamaica, and present at one of the four hospitals for out-patient care or as a visitor. Potential participants were approached by trained research assistants while they were waiting for care at outpatient clinics or utilizing service/public areas at the hospitals (information desk, lobby, corridors). Men who volunteered to participate in the study and satisfied the eligibility criteria went through the informed consent procedure, and were interviewed by a research assistant in private rooms at the hospitals. Each study participant was given a phone card valued at $215 Jamaican dollars (equivalent to US$2.50) after completing the interview. Men who worked at the recruiting hospitals or who were employed by the WRHA were not eligible to participate in this study.

To estimate the potential effect of selection bias in recruiting from hospitals, a sample of 51 men was recruited from a community in the parish of St. James. The hospital sample was compared to the community sample with respect to knowledge of MC (have heard of MC), acceptance of MC (self, infant, son), prevalence of MC, and selected socio-demographic variables (age, income and education). Except for men in the community sample being older, no statistical differences related to other factors were observed between the hospital and community samples. No differences were observed between the parishes with respect to these characteristics.

### Data collection

A 143-item questionnaire was developed to collect data on sexual behaviors, reproductive health practices, male circumcision, health seeking behaviors, attitude towards gender norms and socio-demographic factors. The questionnaire was developed based on an extensive literature review. At the beginning of the interview the men were asked, “have you ever heard about male circumcision?” They were then told the definition of MC. The prevalence of MC was assessed by asking the participants a single question – “Are you circumcised?” Acceptance of male circumcision was assessed by asking three questions: (1) Would you be willing to be circumcised? (2) Would you be willing to consent to having your infant (<1 year) circumcised? (3) Would you be willing to consent to having your son (1-17 years) circumcised? These 3 questions were repeated after an information session in which the men were provided with information about the benefits of MC in reducing men’s risk for HIV infection.

Barriers to MC were assessed by asking participants the following question “What is your greatest concern about MC?” the response choices were: (1) You fear the surgery may damage the penis (2), You do not believe a man should change the way God made the penis (3), You will have to do without sex until the penis heals (4), Other (5), You do not have any concerns about MC. The wording of option 2 – “You do not believe a man should change the way God made the penis” was based on a recommendation from Health Education Officers in the region who vetted the questionnaire before it was used for the study. Health Educators interact on a regular basis with the target population therefore the wording closely reflects the vernacular of the Jamaican people.

Attitudes and beliefs were measured by asking questions relating to MC and hygiene, risk of HIV/STIs, and pleasure during sex. These statements included: (1) “It is harder to keep the penis clean if a man is_ _”, (2) “A man is more likely to experience pain during sexual intercourse if he is_ _”, (3) “A man is likely to enjoy sex more if he is_ _”, and (4) “It is easier for a man to contract HIV if he is_ _”. The response options for these statements were: “circumcised”, “uncircumcised”, “no difference”, and “don’t know”.

Attitude towards masculinity was measured using the Macho Scale which was recently developed by Anderson among fathers (18-59 years of age) residing in Jamaica [38,39]. The Scale [38,39] consists of 13 items (Cronbach’s alpha of 0.82) measuring three dimensions of men’s masculinity related to male-female gender relations. The three dimensions of the scale are the primordial need to produce children, sexual dominance (virility), and domestic freedom. These dimensions are a measure of gender norms regarding masculinity wherein men’s identity is associated with their ability to father children and to have multiple sexual partners. Items were scored using a Likert scale ranging from 1 to 5, resulting in a minimum score of 13 and a maximum of 65. Higher scores were indicative of higher levels of machismo [38,39]. Scores were classified as ‘‘high’’, ‘‘moderate’’ and ‘‘low’’ by dividing the cumulative frequency distribution of the sample into tertiles. One of the items “A man does not have to tell his partner everywhere he is going”, which was included in the version of the Macho scale that was used in this study, has since been replaced by another item “A man should never tell a woman he loves her” in the final scale published by the author [39].

The questionnaire was pretested before it was used in this study.

### Data analysis

The following were calculated: (1) the proportion of men who were circumcised (prevalence), (2) the proportion of men who had heard of MC (awareness), and (3) separately for circumcised and uncircumcised men, the proportions who reported that they would accept MC for themselves, for their male infants (<1 year of age) and their male sons (ages 1-17 years) before being provided educational information on MC. Chi-square tests were used to ascertain statistical significance of associations of each of the above with socio-demographic factors and with attitudes/beliefs regarding MC. Variables (6) assessing beliefs and attitudes towards MC including penile hygiene, pain and pleasure during sex, and ease of contracting HIV/STIs, were dichotomized with circumcised coded as 1 and all the other responses (uncircumcised, no difference, don’t know) coded as 0, before multivariable modeling. This was done because the associations that were observed among responses such as uncircumcised, no difference, and don’t know were very similar. Combining these categories increased interpretability and increased statistical power. Concerns about MC were also dichotomized into any concern or no concern before modeling. Logistic regression was used to ascertain independent associations with awareness and acceptance of MC. Backward selection modeling was used in the adjusted models. Variables with a p-value <0.10 in chi-square analysis were entered into the multivariable logistic regression analyses and retained if p<0.05. The final models for acceptance and knowledge of MC were adjusted for age, income, education, and religion as these were a priori believed to be associated with awareness and acceptance of MC. Adjusted odds ratios (AOR) and 95% confidence intervals (CI) were calculated from the regression equations. Data analysis was performed using SAS software version 9.2 (SAS Institute, Cary, NC).

## Results

Overall, approximately 70% of the men who were approached agreed to participate in the study. The main reasons for not participating were lack of time and conflicting appointment schedule. The mean age of the 549 participants (± standard deviation) was 32.4±10.1 years. Most of the men reported secondary level education (65.4%), skill-based occupation (53.9%), low monthly income (63.4%; median = $30,000 Jamaican Dollars (equivalent to US$349)), single marital status (64.7%) and having some type of religious affiliation (78.6%) (over 30 denominations were identified), almost all were Christians. The majority (>50%) of men reported either not knowing or believing that there were no differences in HIV/STI risk or in pain/pleasure during sex whether or not circumcised. Overall, 9.7% of the men reported “don’t know” to all six questions that evaluated perceptions about MC. Attitudes/beliefs about MC differed significantly between circumcised and uncircumcised men. Notably, among circumcised men 67.5% reported that it would be harder to maintain penile hygiene and easier to contract STIs (44.2%) if the penis is uncircumcised compared to 39.2% and 23.1%, respectively, among uncircumcised men. A substantial proportion (38.7%) of men reported having no concerns about MC. A higher proportion of circumcised men (73.3%) reported no concerns about MC compared to 33.5% of uncircumcised men. The two main concerns about MC reported by uncircumcised men were “Should not change the way God made the penis” (21.2%) and “Surgery may damage the penis” (18.0%). The mean Macho Scale score with standard deviation among the men was 37.6±6 (Table 1).

**Table 1:** Selected characteristics of men in western Jamaica stratified by male circumcision (MC) status.

	All (N=549)			Circumcised (N=77)			Uncircumcised (N=472)		
Selected variables	N	%		N	%		N	%	p-value
**Age (years**)									0.96
19-24	238	43.4		33	42.9		205	43.4	
25-34	154	28.1		21	27.3		133	28.2	
35-54	157	28.6		23	29.9		134	28.4	
**Education**									0.36
Primary or less	118	21.5		16	20.8		102	21.6	
Secondary	359	65.4		47	61.0		312	66.1	
Tertiary	72	13.1		14	18.2		58	12.3	
**Occupation**									0.031
Unskilled	179	35.2		34	44.2		145	33.6	
Skilled	274	53.9		31	40.3		243	56.4	
Professional	55	10.8		12	15.6		43	10.0	
**Monthly Income ($JA**)									0.07
≤30,000	348	63.4		56	72.7		292	61.9	
>30,000	201	36.6		21	27.3		180	38.1	
**Union Status**									0.83
Living together/married	191	35.3		26	34.2		165	35.5	
Single	350	64.7		50	65.8		300	64.5	
**Religion**									0.33
No religion	117	21.4		13	17.1		104	22.1	
Any religion	430	78.6		63	82.9		367	77.9	
**Ever heard about MC^1^**	394	72.2		53^*^	68.0		341	72.9	0.37
**Acceptance of MC for self**									
Yes	-	-		-	-		115	24.5	-
No	-	-		-	-		352	75.4	-
**Harder to keep penis clean if**									**<0.001**
Circumcised	63	11.5		6	7.8		57	12.1	
Uncircumcised	235	43.0		52	67.5		185	39.2	
No difference	119	21.8		8	10.4		111	23.5	
Don’t know	130	23.4		11	14.3		119	25.2	
**Easier to contract STIs if**									**0.001**
Circumcised	65	11.9		5	6.5		60	12.7	
Uncircumcised	143	26.1		34	44.2		109	23.1	
No difference	159	29.1		20	26.0		139	29.5	
Don’t know	181	33.0		18	23.4		163	34.6	
**Easier to contract HIV if**									0.35
Circumcised	39	7.1		7	9.1		32	6.8	
Uncircumcised	65	11.9		13	16.9		52	11.0	
No difference	288	52.5		39	50.7		249	52.9	
Don’t know	156	28.5		18	23.4		138	29.3	
**More pain during sex if**									**<0.001**
Circumcised	44	8.0		6	7.8		38	8.1	
Uncircumcised	117	21.3		30	39.0		87	18.4	
No difference	127	23.2		11	14.3		116	24.6	
Don’t know	261	47.5		30	39.0		231	48.9	
**More pleasure during sex if**									**<0.001**
Circumcised	107	19.6		34	44.2		75	16.0	
Uncircumcised	45	8.3		3	3.9		42	8.9	
No difference	118	21.7		9	11.7		109	23.2	
Don’t know	275	50.5		31	40.3		244	51.9	
**Women enjoy sex more if man is**									**0.003**
Circumcised	76	13.9		20	26.3		56	11.9	
Uncircumcised	24	4.4		5	6.6		19	4.1	
No difference	110	20.2		16	21.1		94	20.0	
Don’t know	335	61.5		35	46.1		300	64.0	
**HIV risk perception**									0.92
Low	400	74.4		55	73.3		345	74.5	
Moderate	67	12.5		9	12.0		58	12.5	
High	71	13.2		11	14.7		60	13.0	
**Ever had an STI** ^2^									0.80
Yes	192	35.0		26	33.8		166	35.2	
No	356	65.0		51	66.2		305	64.8	
**Best age for MC**									0.021
< 1year	219	45.9		42	56.0		177	44.0	
1-17 years	176	36.9		28	37.3		148	36.8	
18 years and older	82	17.2		5	6.7		77	19.2	
**Who prefer to conduct MC**									0.08
Doctor in Public hospital	168	31.4		29	38.7		139	30.2	
Doctor in Private hospital	249	46.5		26	34.7		223	48.5	
It does not matter	118	22.1		20	26.7		98	21.3	
**Greatest concern about MC**									**<0.001**
Surgery may damage penis	90	16.5		5	6.7		85	18.0	
Should not change how God made the penis	103	18.9		3	4.0		100	21.2	
Other	141	25.9		12	16.0		129	27.3	
No concern	211	38.7		55	73.3		158	33.5	
**Macho score**									0.12
Low (13-32^3^)	161	29.3		28	36.4		133	28.2	
Moderate (34-40)	223	40.6		33	42.9		190	40.3	
High (44-65)	165	30.1		16	20.8		149	31.6	

1 MC: Male circumcision (before information session). Only 53 of the 77 men reporting MC, because 24 men had initially reported that they never heard of MC; however, after the definition was given they reported that they were aware of the procedure and that they were circumcised

2 STI: Sexually Transmitted Infection

3 Observed ranges of tertiles of Macho scores

### Prevalence of MC

Seventy-seven (14.0%) men reported that they have been circumcised. Except for occupation (p=0.03), there was no statistical difference between circumcised and uncircumcised men with respect to age (p=0.96), education (p=0.36), income (p=0.07), religion (p=0.33) or marital status (p=0.83) (Table 1).

### Knowledge of MC

Overall, 72.2% of the men in this study reported having heard of MC; 68.0% [53/77] among circumcised (many of these men did not know the terminology, but reported that they were aware after the definition of the procedure was given) and 72.9% [341/472] among uncircumcised men. This knowledge did not differ by age, but was more common among indicators of upper SES (education and occupation), married men who had some type of religious affiliation, men who considered themselves at low risk of HIV acquisition and among men who had low/moderate Macho scores. In the multivariable model, which was adjusted for age, education, religion and income, knowledge of MC was associated with being married (AOR = 1.7; 95% CI =1.0-2.7; p=0.04), having a skilled (AOR =1.5; 95% CI =1.0-2.3; p=0.08) and professional-related occupation (AOR=4.3; 95% CI =1.4-13.1 ; p=0.01) compared to unskilled occupations, and with low (AOR =2.5; 95% CI =1.3-4.4; p=0.003) and moderate (AOR =2.0; 95% CI =1.24-3.2; p=0.005) masculinity scores compared to men with high scores.

Knowledge of MC did not significantly differ by acceptance of MC for self, infants or sons (data not shown.) 

### Acceptance of MC before and after the information session

**Figure 1:**
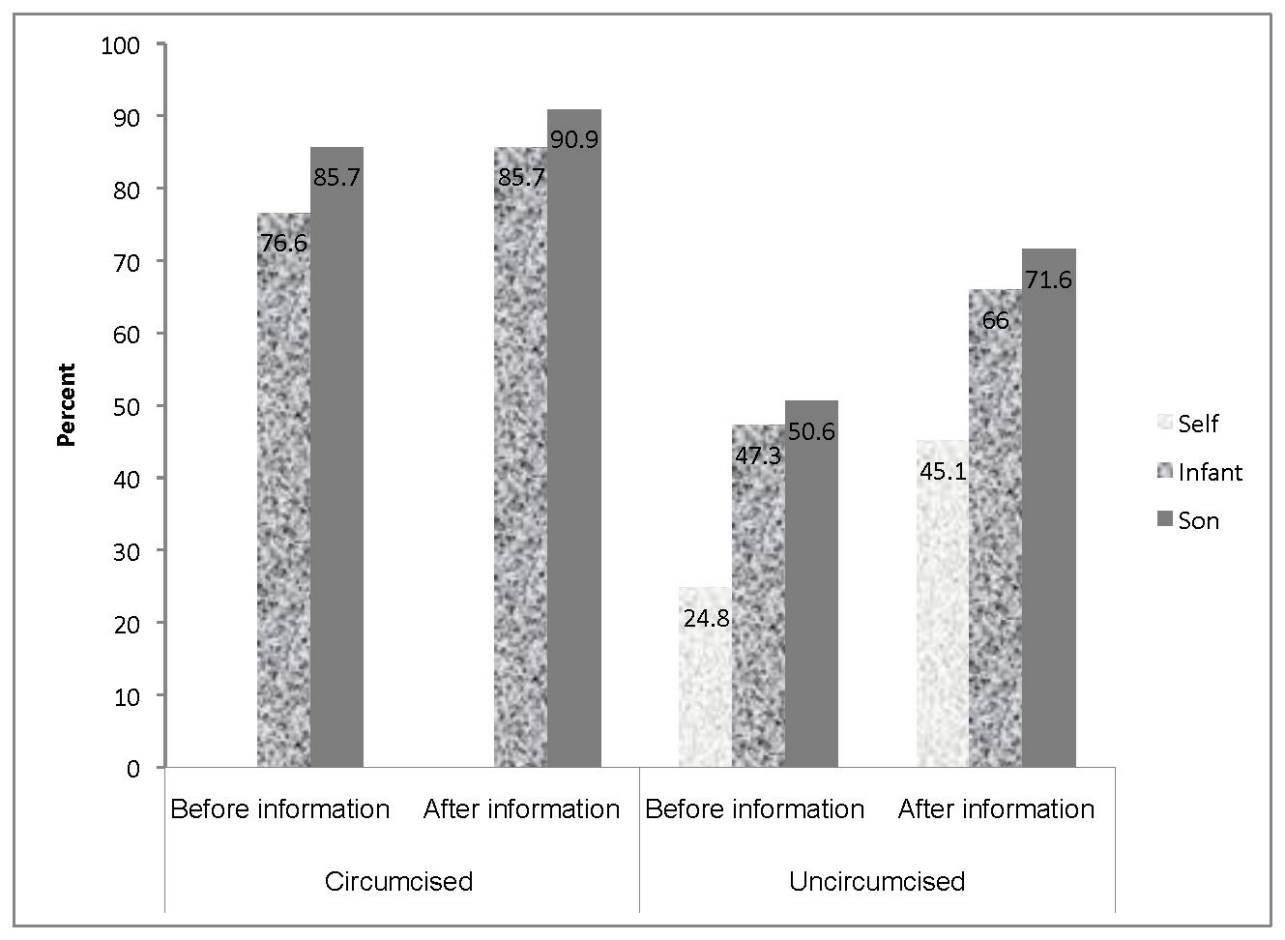
Acceptance of male circumcision among study participants before and after information session stratified by circumcision status

Acceptance of MC for infants and older son swas higher among circumcised than uncircumcised men, was higher for older sons than infants, and increased in all groups after the information session. Before the information session, acceptance of MC among circumcised men was 77% for infants and 86% for sons. These percentages increased to 86% and 91%, respectively, after the information session. Among uncircumcised men, acceptance of MC was 48% for infants and 51% for sons before the information session and increased to 66% and 72%, respectively, after the information session. Among uncircumcised men, acceptance of MC for self, increased from 25% before to 45% after the information session (Figure 1).

### Factors associated with acceptance of MC before the information session

There were no statistically significant differences between circumcised and uncircumcised men with respect to socio-demographic factors and acceptance of MC for infants/sons (Table 2). No statistically significant differences were observed in the beliefs/attitudes towards MC among circumcised men with respect to the acceptance of MC for infants/sons except for their beliefs about penile hygiene. In contrast, many differences in beliefs and attitudes were observed among uncircumcised men (p<0.01 for all differences). Among uncircumcised men, higher proportions who believed it was harder to keep the penis clean (72.1%), easier to contract HIV (82.0%), and that they were more likely to experience more pain during sex (74.1%) if the penis were uncircumcised, reported acceptance of MC for infant/son than men who had different beliefs or reported that they did not know (all were <60%). Also among uncircumcised men, higher proportions who believed more pleasure during sex (82.2%) and that women enjoyed sex more (79.6%) if a man is circumcised, reported acceptance of MC for infant/son than men who had different beliefs or reported that they did not know (all were <62%). Additionally, a higher proportion of uncircumcised men who reported acceptance of MC for themselves tended to report acceptance for their infants/sons. A sizable proportion (>50%), of uncircumcised men reported “no difference” or “don’t know” regarding most of the questions assessing believes/attitudes towards MC (Table 2).

**Table 2:** Frequency of selected characteristics among men accepting (before the information session) of male circumcision (MC) for infant/son stratified by circumcision status (row %).

**Selected variables**		**Acceptance of MC for infants/sons**						
		**Circumcised Men**				**Uncircumcised Men**		
		**N**	**%**	**p-value**		**N**	**%**	**p-value**
**Age (years**)				0.80				0.14
19-24		30	90.9			128	63.1	
25-34		18	85.7			71	53.4	
35-54		21	91.3			73	54.5	
**Education**				0.67				0.14
Primary or less		14	87.5			66	64.7	
Secondary and higher		55	90.2			206	56.0	
**Occupation**								
Unskilled		30	88.2	0.93		85	59.4	0.77
Skilled		28	90.3			135	55.8	
Professional		11	91.7			24	55.8	
**Monthly Income ($JA**)				0.43				0.10
≤30,000		49	87.5			177	60.8	
>30,000		20	95.2			95	53.1	
**Union Status**				0.71				0.07
Single		24	92.3			105	63.6	
Living together/married		44	88.0			164	55.0	
**Religion**				0.34				0.43
No religion		11	84.6			56	54.4	
Any religion		58	92.1			215	58.7	
**Ever heard about MC**		49	90.7	0.71		199	58.5	0.62
**Acceptance of MC for self**		-	-	-				**<0.001**
Yes		-	-			102	90.3	
No		-	-			166	47.3	
**Harder to keep penis clean if**				**0.032**				**<0.001**
Circumcised		6	100.0			27	47.4	
Uncircumcised		49	94.2			132	72.1	
No difference		7	87.5			57	51.4	
Don’t know		7	63.6			56	47.1	
**Easier to contract STIs if**				0.17				**0.005**
Circumcised		3	60.0			31	51.7	
Uncircumcised		32	94.1			81	75.7	
No difference		18	90.0			73	52.5	
Don’t know		16	88.9			87	53.4	
**Easier to contract HIV if**				0.17				**0.002**
Circumcised		5	71.4			17	53.1	
Uncircumcised		13	100.0			41	82.0	
No difference		36	92.3			143	57.4	
Don’t know		15	83.3			70	50.7	
**More pain during sex if**				0.55				**0.005**
Circumcised		6	100.0			21	55.3	
Uncircumcised		28	93.3			63	74.1	
No difference		9	81.8			68	58.6	
Don’t know		26	86.7			120	52.0	
**More pleasure during sex if**				0.19				**<0.001**
Circumcised		32	94.1			60	82.2	
Uncircumcised		2	66.7			21	50.0	
No difference		9	100.0			57	52.3	
Don’t know		26	83.9			132	54.1	
**Women enjoy sex more if man is**				0.53				**0.004**
Circumcised		19	95.0			43	79.6	
Uncircumcised		4	80.0			10	52.6	
No difference		15	93.8			58	61.7	
Don’t know		30	85.7			161	53.7	
**HIV risk perception**				0.67				0.17
Low		49	89.1			207	60.4	
Moderate		8	88.9			31	53.5	
High		11	100.0			29	48.3	
**Ever had an STI^1^**				0.99				0.28
Yes		46	90.2			101	61.2	
No		23	88.5			170	55.9	
**Best age for MC**				**<0.001**				**<0.001**
< 1year		40	95.2			133	72.7	
1-17 years		28	93.3			100	69.0	
18 years and older		2	33.3			21	29.2	
**Who prefer to conduct MC**				0.99				0.07
Doctor in Public hospital		26	89.7			92	66.2	
Doctor in Private hospital		23	88.5			119	53.9	
It does not matter		18	90.0			58	21.6	
**Greatest concern about MC**				0.14				**<0.001**
Surgery may damage penis		4	80.0			47	55.3	
Should not change how God made penis		2	66.7			30	30.0	
Other		10	83.3			75	58.1	
No concern		51	68.0			120	76.9	
**Macho score**				0.71				0.84
Low (13-32)		24	85.7			79	59.9	
Moderate (34-40)		30	90.9			109	57.7	
High (44-65)		15	93.8			84	56.4	

1 STI: Sexually Transmitted Infection

In the multivariable model, the analysis was adjusted for age, education, religion, and income, the variables that remained significant in the model were beliefs/attitudes about men’s pleasure during sex, concerns about MC, and acceptance of MC for self. There were increased odds of accepting MC for infants/sons among uncircumcised men who accepted MC for self (AOR=8.1; 95% CI = 4.1-15.9), believed they would experience more pleasure during sex if circumcised (AOR=4.0; 95% CI = 2.0-8.2), and reported having no concerns regarding MC (AOR=3.0; 95% CI = 1.8-4.8) (Table 3).

**Table 3:** Multivariable analysis of factors associated with acceptance of male circumcision (MC) for infants/sons and sons among uncircumcised men.

**Factors**	**Acceptance of MC for infant/son**		**Acceptance of MC for self**	
	**Adjusted^1^ Odds Ratio (95% CI^2^**)	**P-value**	**Adjusted^1^ Odds Ratio (95% CI^2^**)	**P-value**
**Age (years**)				
19-24	0.9 (0.5–1.5)	0.40	0.7 (0.5-1.5)	0.41
25-34	0.8 (0.5-1.5)	0.79	0.9 (0.5-1.5)	0.65
35-54	Referent			
**≥Secondary education**	0.8 (0.5-1.4)	0.49	1.0 (0.6-1.7)	0.91
**Income >30,000**	0.7 (0.4-1.1)	0.12	0.9 (0.5-1.4)	0.52
**Any religion**	1.1 (0.6-1.8)	0.79	1.3 (0.8-2.4)	0.31
**Accept MC for self**	**8.1 (4.1-15.9)**	**<0.001**	**-**	**-**
**More pleasure during sex if**				
Circumcised	**4.0 (2.0-8.2**)	**0.002**	**2.7 (1.5-4.5)**	**0.004**
**No concern about MC**	**3.0 (1.8-4.8)**	**<0.001**	**2.6 (1.7-4.1)**	**<0.001**

1 All characteristics listed were adjusted for

2 CI: Confidence interval

We constructed a model using the same variables as described in Table 2 to determine the factors associated with acceptance of MC for self among uncircumcised men. In the multivariable model, which adjusted for age, religion, education and income, we observed similar associations to those we found with acceptance for infant/son. Increased odds of accepting MC for self among uncircumcised men was associated with beliefs about experiencing more pleasure during sex if circumcised and reporting having no concerns regarding MC (Table 3).

## Discussion

International agencies such as the WHO and UNAIDS recommend MC in HIV prevention programs for countries with low prevalence of MC, high rates of HIV infection, and epidemics driven by heterosexual sex, based on overwhelming evidence showing that MC can reduce heterosexual transmission by approximately 60% [1,5-9,40]. However, decisions to translate research into public health policy and practice can be very challenging for some countries due to concerns about unanticipated consequences, conflict of cultural beliefs, and lack of support from stakeholders including politicians. For Caribbean countries like Jamaica, in which MC is not traditionally performed [17], it is imperative to understand the attitude of individuals towards MC and the factors associated with acceptance to guide policy and program implementation. We conducted a cross-sectional study among men in western Jamaica to identify the factors that were associated with acceptance of MC, and found that acceptance of MC varied by the MC status of men, and was more favorable for infants and older sons than for self.

Overall, the relatively high levels of acceptance of MC for infants and sons are promising given that MC is not routinely performed in Jamaica [17]. While providing MC during infancy and older childhood years will not immediately impact the rate of heterosexual transmission of HIV as would an adult MC program [7], we believe investment in an infant/childhood MC program is crucial for Jamaica to effectively impact the HIV epidemic in the future. Findings from cost benefit analysis of infant MC in the US [41] and Rwanda [42] revealed that it is a cost-effective measure for reducing HIV acquisition. Risk behaviors, such as multiple sexual partners, especially among youth [27,34,43], low condom use at last sex [34] and early sexual debut (sexual initiation by the age of 14) [44,45], which are prevalent in Jamaica, underscore the need to include MC in the country’s HIV prevention program. Although MC is one of the oldest and most common of surgical procedures [46], and has been proven to be cost effective and efficacious in reducing the risk of HIV acquisition among heterosexual men [1-4], its public health importance is still highly debated in both developing and developed countries [46,47]. Some may argue that there is no need to implement MC in countries that do not meet the criteria set out by the WHO and UNAIDS for including MC in HIV prevention [1,5-9,40]. However, an argument could also be made that it may be necessary for the WHO/UNAIDS to revisit its recommendations to determine the worth of expanding the criteria to include countries with relatively low HIV prevalence with special emphasis on promoting neonatal MC. This would be justified in light of the overwhelming evidence that MC is efficacious in reducing HIV acquisition and has a low risk of adverse effects especially if conducted during the neonatal period. The HIV epidemic has thrived in many countries because of a delayed response to HIV prevention. Therefore, we should endeavor to be proactive and use all the tools that are available to effectively halt this pandemic.

We found similar acceptance levels of MC for self among uncircumcised men (25%) as that reported in Figueroa’s study (23%) [31]. However, in our study acceptance of MC for self, increased to 47% after the information session, suggesting that knowledge had a positive effect on the reported intention to accept MC. A similar increase has been observed in another Caribbean country (Dominican Republic) where acceptance of MC for self was 29% before the information session but increased to 67% after the inforamtion session [21]. . Overall, knowledge of MC among men in this study appeared to be low as a significant proportion of men reported “don’t know” on a number of the questions relating to attitude/beliefs about MC. Further, approximately 28% of the participants reported they had never heard of MC prior to the study. However, after the definition of MC was given a number of the men reported that they were aware of the procedure but not the terminology. This may explain why 24 of the 77 men who reported being circumcised stated that they had never heard of MC prior to the study. The prevalence of MC among men in this study was 14%, which is a bit higher than the 9% found in Figueroa’s study. However, if the 24 circumcised men who reported that they had not heard of MC prior to the study were excluded, the prevalence of MC among men in this study would be 9.8%. The difference in MC prevalence in our study and Figueroa’s may be due to a lack of understanding of the term “MC” in Figueroa’s study, possible over reporting of MC in our study, geographical differences or selection bias (Figueroa’s study was conducted among clients at an STI clinic in the capital city of Jamaica while our study was conducted among outpatient clients and visitors at hospitals in the western region of Jamaica).

Of note, our findings did not reveal any association between acceptance of MC and support of masculinity norms. This was surprising, but may be due to the fact that the Macho Scale primarily seeks to measure dimensions of masculinity relating to sexual dominance, virility, and domestic freedom, rather than disease prevention. Beliefs relating to HIV/STI acquisition [7,18,20,21,23,48], and penile hygiene [18,21,49], which are well established in the literature, were not associated with acceptance of MC among men in this study. This may be indicative of the limited knowledge about MC among the men. In this study beliefs about pleasure during sex and acceptance of MC for self [10,50,51], which are consistent with findings in the literature, were associated with acceptance of MC. Additionally, men who did not have any concerns about MC were more likely to accept it. Thus, MC educational and promotional programs must address men’s attitude towards MC as well as beliefs regarding “changing the way God made the penis” and effects of the surgery resulting in damage to the penis, as these could serve as significant barriers to the uptake of MC.

Although our study provides a better understanding of the factors associated with the acceptability of MC among men in Jamaica, a country in which MC is not traditionally performed, there are some limitations that must be considered in the interpretation the results. First, the study depended solely on self-reported data via interviewer administered questionnaires. Thus, the findings could be influenced by social desirability bias especially as it relates to reporting the prevalence of MC (there may be over reporting). Although, all the interviewers were trained to reduce this potential bias, we are unable to estimate its effects on the study. Second, the participants were recruited at hospitals, hence the study is subject to selection bias. Although we included visitors at the participating hospitals to moderate this potential bias, we did not document what proportion of the participants were visitors. However, we compared the hospital sample to a small community sample with respect to the outcome variables and key socio-demographic factors and found no difference between the two samples expect for age. This suggests the effect of selection bias in our sample may be minimal. Third, we did not use a probability sampling technique to recruit participants.

In spite of these limitations, the findings of our study suggest that men who reside in the western region of Jamaica are likely to be receptive to MC for their infants and older sons as an HIV prevention strategy, especially if the program is bolstered with an effective health education component. Fathers play a crucial role in decisions regarding MC for their infants [51,52]; however, men are often excluded from health related decisions involving their children [53,54]. Thus, the beliefs and attitudes of Jamaican men towards MC as documented in this study could inform policy decisions about the implementation of a MC program in Jamaica. Because there is still disagreement among policy makers and health care workers in the Caribbean region about the effectiveness of MC as an HIV prevention strategy [17] it would be prudent for further research to be conducted to assess the attitudes of health workers towards MC as well as their perceived ability to safely conduct MC, and the feasibility of including MC in Jamaica’s health care system. Irrespective of the decision to include MC in HIV prevention programs, it is pivotal to educate men about MC so that they can make an informed decision and choose MC for their sons if so desired. Taking measures such as hosting public debates, media promotions, and partnering with men and community-based organizations to sensitize the public on the effectiveness of MC as an HIV prevention strategy would be beneficial for Jamaica and other Caribbean countries.
